# Integrating Multimodal MRI Habitat and Transformer‐Based Pathomics to Predict High‐Risk Molecular Subtypes and Explore Biological Mechanisms in Adult Diffuse Gliomas

**DOI:** 10.1002/cns.71171

**Published:** 2026-09-28

**Authors:** Wenju Niu, Xin Duan, Xuan Li, Zehui Li, Qian Liang, Xiangli Yang, Yan Tan, Xiaochun Wang, Guoqiang Yang, Hui Zhang

**Affiliations:** ^1^ Department of Radiology First Hospital of Shanxi Medical University Taiyuan Shanxi Province China; ^2^ College of Medical Imaging Shanxi Medical University Taiyuan Shanxi Province China; ^3^ Third Hospital of Shanxi Medical University, Shanxi Bethune Hospital, Shanxi Academy of Medical Sciences, Tongji Shanxi Hospital Taiyuan China; ^4^ Shanxi Key Laboratory of Intelligent Imaging and Nanomedicine First Hospital of Shanxi Medical University Taiyuan Shanxi Province China; ^5^ Intelligent Imaging Big Data and Functional Nano‐Imaging Engineering Research Center of Shanxi Province First Hospital of Shanxi Medical University Taiyuan Shanxi Province China

**Keywords:** deep learning, glioma, habitat, MRI, transformer

## Abstract

**Background:**

This study aims to achieve accurate prediction of high‐risk molecular subtypes of gliomas through a cross‐scale Combined model, matching the model's classification metrics with patient risk stratification and exploring the underlying biological mechanisms.

**Methods:**

This study retrospectively collected preoperative MRI, postoperative whole‐slide pathological images, molecular markers, and clinical data from 456 adult diffuse glioma patients. We separately constructed an MRI habitat prediction model, a WSI Transformer‐based deep learning pathomics (PDL) model, and a Combined model. A dynamic nomogram web page for predicting high‐risk molecular subtypes was developed based on the Combined model. Patients were stratified into risk groups according to the output scores of the Combined model, and Kaplan–Meier survival analysis and the Log‐rank test were employed to evaluate survival differences between the groups. Additionally, differential expression and GO/KEGG enrichment analyses were further performed in the test set with available RNA‐seq data to explore transcriptional features and biological processes associated with model‐based risk stratification.

**Results:**

The Combined model demonstrated the highest AUC (Training set: 0.888, Test set: 0.836) compared to the Habitat model (Training set: 0.832, Test set: 0.798) and the PDL model (Training set: 0.852, Test set: 0.821). The high‐risk and low‐risk groups, stratified based on the cutoff value derived from the Combined model output scores, exhibited significant survival differences. Exploratory transcriptomic analysis showed that differentially expressed genes between the high‐ and low‐risk groups were mainly enriched in biological processes and pathways related to the extracellular matrix and cell–matrix interactions.

**Conclusion:**

The cross‐scale Combined model not only enabled identification of high‐risk molecular subtypes and risk stratification but also showed associations with biologically relevant transcriptional features.

## Graphical abstract

Based on the 2021 WHO CNS5 guidelines, this study introduces an integrated high‐risk molecular classification for gliomas and constructs a predictive model by combining multimodal MRI habitat imaging, Transformer‐based WSI pathomics, and clinical information.

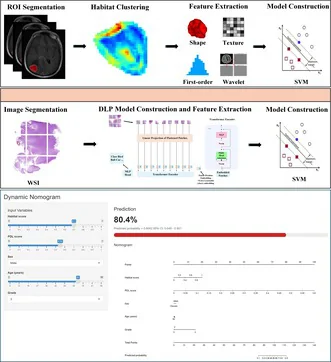

## 1 Introduction

Gliomas, as the most aggressive primary tumors of the central nervous system (CNS), possess complex molecular heterogeneity, which serves as the core factor driving malignant progression, treatment resistance, and poor prognosis of the tumors [1, 2]. The pivotal innovation in the 2021 WHO Classification of Tumors of the Central Nervous System lies in the establishment of the “integrated molecular diagnosis” paradigm for gliomas, identifying CDKN2A/B homozygous deletion, TERT promoter mutation, EGFR amplification, and + 7/−10 chromosomal alterations as high‐risk molecular markers for gliomas [3]. These molecular markers not only can independently predict the malignant biological behaviors of gliomas [4] but have also been confirmed to directly lead to an upgrade in tumor grading beyond traditional histological classification [3]. However, the malignant progression of high‐risk molecular subtypes in gliomas cannot be attributed to a single molecular mechanism; rather, it is the result of the interaction and concerted evolution of multiple molecular mechanisms. For instance, while TERT promoter mutations correlate with favorable prognosis in IDH‐mutant gliomas [5], they are associated with enhanced aggressiveness and worse outcomes in IDH‐wildtype tumors [6]. This intricate molecular network heterogeneity, coupled with complex interactions within the tumor microenvironment, collectively shapes the invasive phenotype and clinical trajectory of gliomas [7, 8]. Therefore, comprehensive characterization of glioma molecular heterogeneity and microenvironmental complexity is essential for accurate identification of high‐risk molecular subtypes.

In clinical practice, the identification of high‐risk molecular subtypes of gliomas faces dual challenges: On one hand, the gold standard relies on invasive tissue biopsy and multiple molecular tests, which not only increase the burden on patients but may also lead to sampling errors due to the spatial heterogeneity of tumors [9]. On the other hand, while conventional imaging can provide macroscopic structural information, its ability to analyze molecular characteristics and microenvironmental heterogeneity is limited [10]. Traditional radiomics analysis methods often treat tumors as homogeneous entities, overlooking the ecosystem properties of tumors composed of subregions (habitats) with distinct pathophysiological features [11, 12]. Emerging habitat imaging technology, rooted in the concept of Darwinian evolutionary dynamics, establishes interpretable associations between macroscopic imaging manifestations and microscopic molecular features by segmenting tumors into subregions and quantifying the radiomic characteristics of different habitats [13, 14, 15], thereby opening up new avenues for non‐invasive analysis of tumor spatial heterogeneity.

Concurrently, computational pathology for artificial intelligence (AI)‐assisted analysis is rapidly advancing. Pathomics analysis based on Whole Slide Image (WSI) enables the quantification of tumor micro‐heterogeneity, facilitating the prediction of molecular status [16, 17] and prognosis [18] in gliomas. Research indicates that transformer‐based deep learning models exhibit unique advantages in processing large‐scale WSIs, showing remarkable potential for predicting biomarkers across various tumor types [19, 20, 21]. However, existing studies have predominantly focused on single‐scale analyses, failing to fully integrate the complementary cross‐scale information between macroscopic habitat features and microscopic pathological characteristics. This limitation has constrained the comprehensive characterization of glioma multidimensional heterogeneity and optimization of predictive performance.

Therefore, this study aimed to integrate macroscopic MRI habitat imaging, microscopic WSI analysis based on Transformer‐driven deep learning, and pivotal clinical information to construct a cross‐scale model for the accurate prediction of high‐risk molecular subtypes in gliomas. Furthermore, we sought to correlate the model's classification metrics with patient risk stratification and to explore the transcriptional features potentially associated with the model‐derived risk stratification. This is crucial for understanding the heterogeneity of gliomas and may inform the development of personalized treatment strategies. This cohort study has been reported in line with the STROCSS guidelines.

## 2 Materials and Methods

### 2.1 Patient Population and Data Acquisition

The research flowchart is shown in Figure 1. This multicenter retrospective study enrolled 456 adult‐type diffuse gliomas. It comprised 323 consecutive cases from the First Hospital of Shanxi Medical University, Shanxi Provincial People's Hospital, and Shanxi Bethune Hospital between January 2011 and September 2023, along with 133 cases from The Cancer Genome Atlas (TCGA) database. The inclusion criteria were as follows: (1) availability of key molecular markers (including IDH1/2 mutation, 1p/19q codeletion, TERT promoter mutation, EGFR amplification, +7/−10 chromosomal copy number changes, and CDKN2A/B homozygous deletion) to confirm diagnosis based on the 2021 classification criteria; (Figure 2) (2) complete preoperative contrast‐enhanced T1‐weighted (T1CE) and T2‐weighted fluid‐attenuated inversion recovery (T2FLAIR) images; (3) accessible WSIs. For TCGA cases, an additional requirement was available for paired RNA sequencing (RNA‐seq) data. Exclusion criteria comprised: (1) incomplete T1CE or T2FLAIR images; (2) suboptimal WSI quality (including inadequate tissue fixation, poor staining quality, etc.); (3) missing key clinicopathological or molecular data; (4) concurrent central nervous system disorders (e.g., cerebral infarction, hemorrhage, or Parkinson's disease). Overall survival (OS) was defined as the duration from pathological diagnosis to death or last follow‐up. The flowchart for patient selection is shown in Supporting Information 3. The list of patients included from TCGA is detailed in the Supporting Information 1.

**FIGURE 1:**
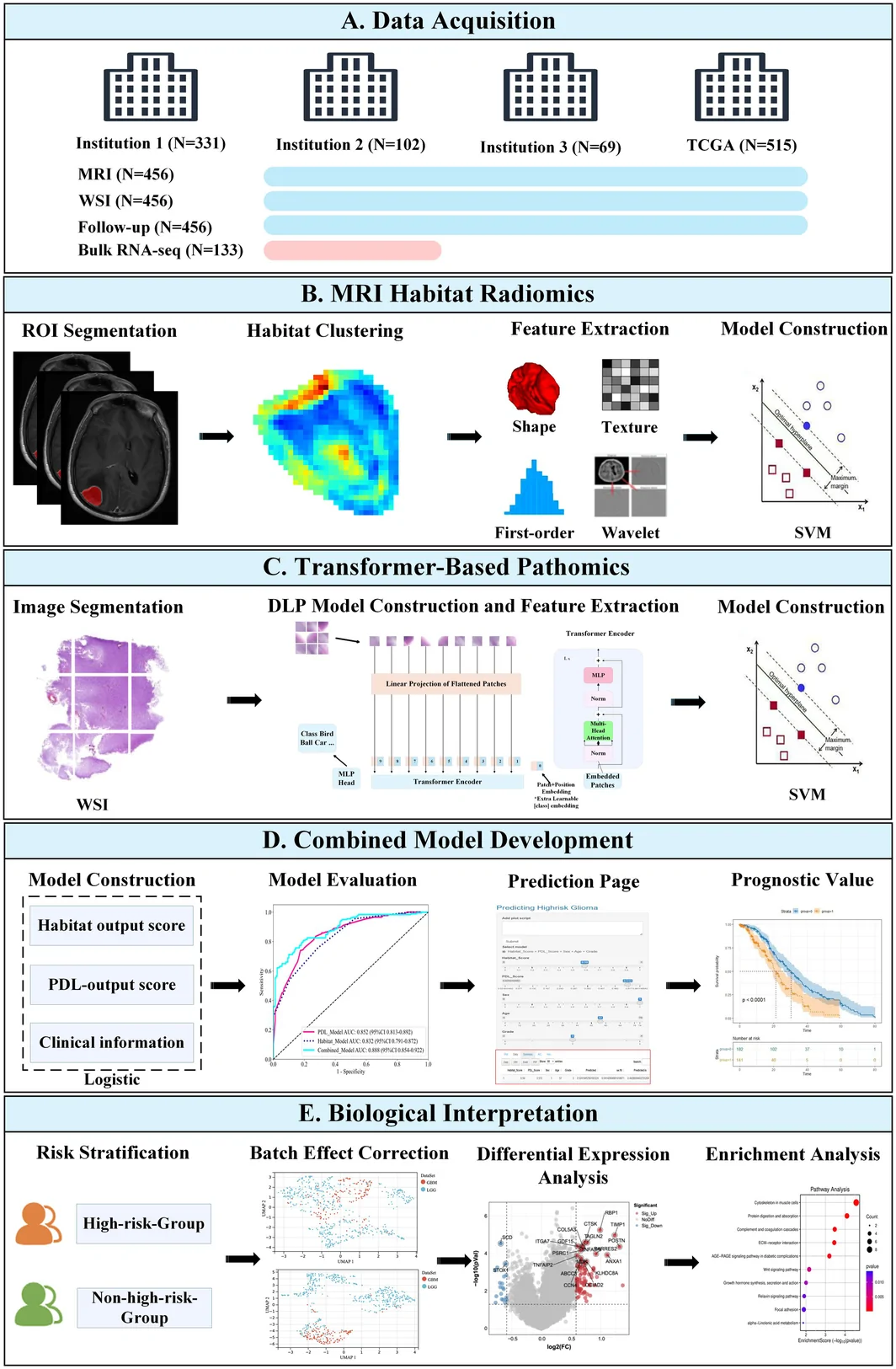
The research flowchart.

**FIGURE 2:**
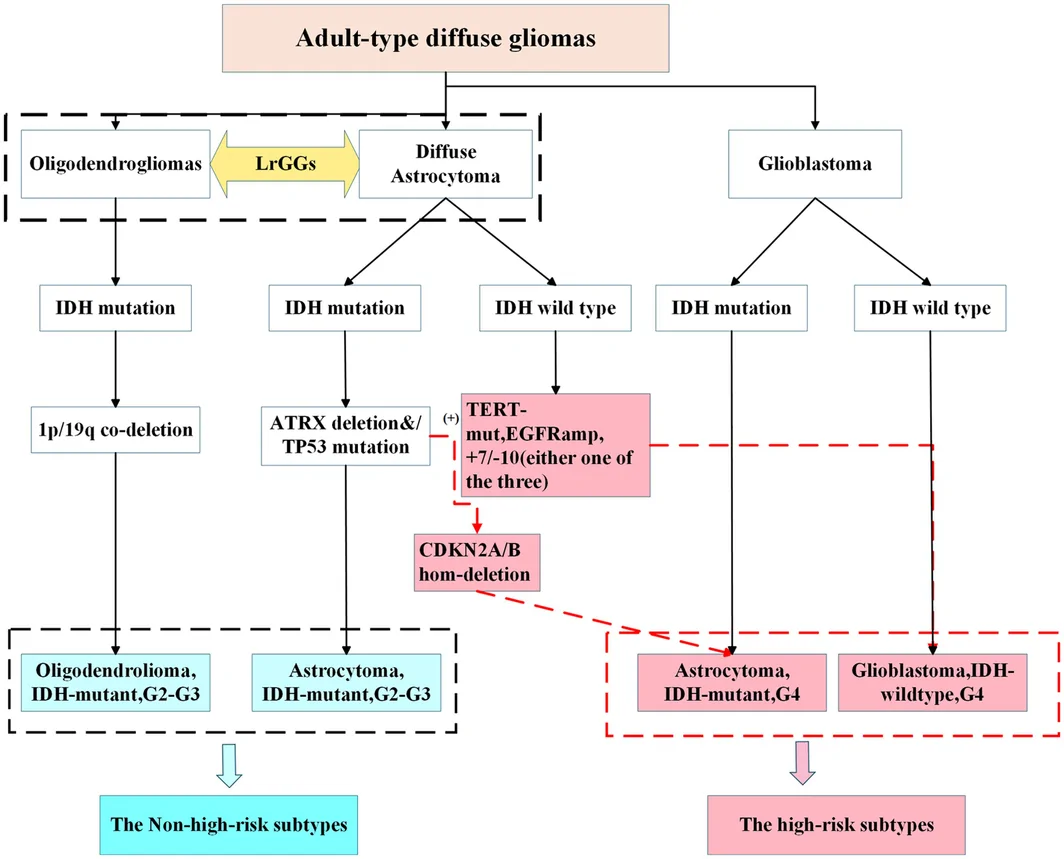
The schematic diagram of adult diffuse glioma classification in this study. IDH: Isocitrate dehydrogenase;1p/19q: Short arm of chromosome 1 and long arm of chromosome 19; TERTp‐mut: Telomerase reverse transcriptase promoter mutation; EGFRamp: Epidermal growth factor receptor amplification; +7/10: Chromosome 7 gain and chromosome 10 loss; CDKN2A/B hom‐deletion: Cyclin‐dependent kinase inhibitor 2A/B homozygous deletion; G2/3/4: Grade2/3/4.

### 2.2 High‐Risk Molecular Marker Detection

In this study, the high‐risk molecular subtype was defined according to the 2021 WHO CNS5 criteria as follows: IDH‐wildtype glioblastomas and IDH‐mutant astrocytomas with CDKN2A/B homozygous deletion were classified as the high‐risk group; the remaining cases (IDH‐mutant astrocytomas grades 2/3, oligodendrogliomas, etc.) were classified as the non‐high‐risk group (Figure 2). This binary label served as the standard prediction target for all subsequent model development and evaluation. To determine the IDH, CDKN2A/B, and TERTp status of gliomas, the Simlex OUP FFPE DNA extraction kit (TIB, Shanghai, China) was utilized following standardized DNA extraction procedures. Subsequently, glioma DNA was PCR amplified using the ABI 9700 Life Technology system (Thermo Fisher Scientific, Waltham, MA, USA), and final detection results were obtained through Sanger sequencing performed on the ABI 3500 Life Technology platform (Thermo Fisher Scientific, Waltham, MA, USA). Additionally, the FISH method was employed to determine EGFR amplification status and chromosome 7/10 alterations, and final detection results were obtained using the probe reagent kit (VividFISH FISH CEP, GeneCopoire).

### 2.3 MRI Image Processing and Segmentation

All preoperative MRI examinations were performed using 1.5 T or 3.0 T scanners detailed scanning parameters of the three local cohorts are provided in Supporting Information 2. We employed the T1CE as the reference image to perform rigid spatial registration on the T2FLAIR sequences. To mitigate resolution variations caused by different scanning equipment and protocols, all imaging data were resampled to a uniform size of 1 × 1 × 1 mm [3]. The regions of interest (ROIs) were meticulously delineated on T1CE sequences by an experienced neuroradiologist (with over 10 years of specialized practice) using ITK software (version 3.6.0), with careful exclusion of peritumoral edema. To ensure annotation reliability, the delineations were independently reviewed by a senior expert (with over 15 years of experience) who verified the results and provided corrective recommendations when necessary. Both readers were blinded to clinical and pathological data. This two‐tiered quality control process was implemented to ensure segmentation reliability.

### 2.4 Tumor Habitat Analysis and Feature Extraction

In this study, we employed the K‐means clustering algorithm to partition tumor regions based on multiparametric MRI data automatically. By integrating the segmentation mask information from T1CE and T2FLAIR sequences, we extracted 15 radiomic features for each voxel as local characterization metrics. Clustering analysis utilized Euclidean distance to measure similarity, and we evaluated clustering schemes with 2 to 10 clusters at the cohort level. To ensure robustness, each cluster configuration underwent 100 iterative computations, and the optimal 3‐cluster solution was determined by maximizing the average Calinski‐Harabasz score, which effectively characterized heterogeneity while avoiding excessive model complexity. Following the Imaging Biomarker Standardization Initiative (IBSI) guidelines, 1743 radiomic features were systematically extracted from each habitat region using the PyRadiomics package (https://github.com/Radiomics/pyradiomics). These comprised: (a) 14 shape‐related features; (b) 342 first‐order statistical features; (c) 266 features derived from the gray‐level dependence matrix (GLDM); (d) 304 features from the gray‐level size zone matrix (GLSZM); (e) 95 features based on the neighboring gray‐tone difference matrix (NGTDM); (f) 304 features from the gray‐level run‐length matrix (GLRLM); and (g) 418 features from the gray‐level co‐occurrence matrix (GLCM). A total of 10,458 radiomic features were extracted from the habitat regions of two sequences.

### 2.5 WSI Preprocessing

The enormous size and ultra‐high resolution of WSI pose significant challenges for computational pathology algorithms. To address this issue, we split each WSI into non‐overlapping 512 × 512‐pixel patches (corresponding to 20× magnification). Each patch underwent stain normalization via the Vahadane method, which projects its optical density‐derived stain composition onto a reference stain matrix through non‐negative matrix factorization, ensuring inter‐specimen staining consistency while preserving tissue morphology integrity.

### 2.6 Deep Learning Pathomics Model Construction and Feature Extraction

We developed a deep learning pathomics (PDL) model built upon a Vision Transformer (ViT) architecture with an embedding dimension of 1024, 6 layers, and 16 attention heads. Model parameters were optimized using stochastic gradient descent (SGD) with a batch size of 128 and L2 regularization. The model was trained for 50 epochs with an initial learning rate of 0.01. For feature aggregation, we adopted two multi‐instance learning (MIL) paradigms: The Patch Likelihood Histogram (PALHI) method based on probability distributions and the Bag of Words (BoW) model based on statistical features. Specifically, PALHI constructs WSI representations by analyzing patch prediction probability distributions, while BoW leverages the TF‐IDF weighting scheme to compute codeword statistics. Ultimately, a feature‐level fusion strategy was adopted to combine the strengths of both approaches, producing discriminative WSI‐level representations. The architecture diagram of the Vision Transformer is presented in Supporting Information 4.

### 2.7 Feature Selection and Model Development

In the process of feature selection, we treated habitat radiomic features and deep‐learning pathomics features separately. Before feature selection, all habitat radiomic features and deep‐learning pathomics features were standardized using Z‐score normalization. The normalization parameters were determined exclusively from the training set and then applied to the test set. Initially, to ensure feature reproducibility, we calculated the Intraclass Correlation Coefficient (ICC) and retained only those features with an ICC greater than 0.80. For each category of features, we conducted Mann‐Whitney U tests. Only features with a *p*‐value below 0.05 were retained. Subsequently, we computed Spearman's rank correlation coefficients among the highly replicable features to evaluate their mutual dependencies. When the correlation coefficient between any pair of features exceeded 0.9, we kept just one of them. To further boost the descriptive capacity of the features, we adopted a greedy recursive elimination approach for feature refinement. This involved iteratively removing the most redundant feature. Finally, we employed the Least Absolute Shrinkage and Selection Operator (LASSO) regression method to pinpoint the optimal feature subset. This subset consisted of crucial features with non‐zero coefficients, selected based on the optimal tuning parameter λ, which was determined via 10‐fold cross‐validation on the training set.

After the LASSO‐based feature selection, the final subsets of the two types of features were input into the SVM machine learning model to construct the Habitat and PDL models, respectively. Output scores from the single‐modality model and clinical information (age, sex, histologic grade) were collected and used to create a Combined model with logistic regression.

### 2.8 Model Performance Evaluation and Development of a Visualization and Prediction Page

To bolster the robustness of our models, we executed the entire construction process 1000 times using the bootstrapping method. Subsequently, the model that exhibited the best performance in the training set was selected for validation on the test set. Visual assessments of the models' predictive capabilities were conducted using Receiver Operating Characteristic (ROC) curves, calibration curves, and Decision Curve Analysis (DCA). Additionally, diagnostic metrics for the three single‐modality models and the Combined model were computed, encompassing the Area Under the Curve (AUC), 95% confidence interval (95% CI), specificity, sensitivity, accuracy, Positive Predictive Value (PPV), and Negative Predictive Value (NPV). In addition, an online version of the nomogram was created to enable clinicians to determine the probability of a patient having a high‐risk subtype of glioma by inputting relevant features (https://gliomaresearch.shinyapps.io/GliomaNomogram/).

### 2.9 Quantitative Evaluation of the Prognostic Value of the Combined Model

The cutoff value for risk stratification was determined based on the median of the Combined model output scores in the training set, and this same cutoff was then applied to the test set for risk group classification. Subsequently, we uniformly divided the study cohort into high‐risk and low‐risk groups. To evaluate whether the output scores of the Combined model could serve as a valid prognostic indicator, we performed Kaplan–Meier survival analysis and the Log‐rank test. These analyses were conducted to examine and compare the survival disparities between the high‐risk and low‐risk groups.

### 2.10 Transcriptomic Analysis of Combined Model‐Based Risk Groups

First, the ComBat algorithm was employed to remove batch effects from the TCGA test set. The gene expression matrix of 133 patients was extracted from the total matrix after batch effect removal. For the high‐risk and low‐risk groups classified based on the output scores of the Combined model, differential expression analysis was conducted using the limma package. The fold change was set to be greater than or equal to 1.5, and the *p*‐value was set to be less than 0.05. Subsequently, Gene Ontology (GO) and Kyoto Encyclopedia of Genes and Genomes (KEGG) enrichment analyses were performed on the differentially expressed genes. GO analysis included Biological Process (BP), Cellular Component (CC), and Molecular Function (MF), and the top 10 enriched terms in each GO category and KEGG were displayed.

### 2.11 Statistical Analysis

When dealing with continuous variables that exhibited homoscedasticity, we opted for either the Mann–Whitney U test or the independent *t*‐test. For categorical variables presented as ratios, we chose either the Chi‐square test or Fisher's exact test. The “Survival” package in R was utilized to conduct Kaplan–Meier survival analysis. All statistical analyses were conducted using the R programming language (version 4.2.2) and the scikit‐learn library (version 0.18) within Python 3.7. All hypotheses were tested using a two‐sided approach, and statistical significance was defined as a two‐tailed *p*‐value of less than 0.05.

## 3 Results

### 3.1 Patient Characteristics Analysis

Table 1 displays the baseline characteristics of the patients enrolled in this study. A total of 456 glioma patients were incorporated into the research. In the training set, 54.80% (177/323) of the patients were classified as having the high‐risk molecular subtype, while in the test set, 48.87% (65/133) of the patients fell into this category. Both age and histologic grade demonstrated significant associations with the high‐risk and non‐high‐risk molecular subtype groups in both the training and test sets. Nevertheless, sex did not show a significant correlation with the high‐risk and non‐high‐risk molecular subtype groups in either the training or test sets. The patient baseline table is presented in Table 1.

**TABLE 1:** Baseline characteristics of patients in the study sets.

Characteristics	Training set (*n* = 323)		*p*‐value	Test set (*n* = 133)		*p*‐value
	The high‐risk subtypes (*n* = 177)	The non‐high‐ risk subtypes (*n* = 146)		The high‐risk subtypes (*n* = 65)	The non‐high‐ risk subtypes (*n* = 68)	
Age (years)	51.50 ± 15.07	45.38 ± 15.49	< 0.001*	53.91 ± 12.83	43.35 ± 13.82	< 0.001*
Sex			1			0.436
Male	87 (49.15)	72 (49.32)		35 (53.85)	31 (45.59)	
Female	90 (50.85)	74 (50.68)		30 (46.15)	37 (54.41)	
Histologic grade			< 0.001*			< 0.001*
2	37 (20.90)	80 (54.79)		11 (16.92)	44 (64.71)	
3	53 (29.94)	58 (39.73)		37 (56.92)	24 (35.29)	
4	87 (49.15)	8 (5.48)		17 (26.15)	Null	

*Note: p*‐value < 0.05.

* Were considered significant differences.

### 3.2 Habitat Clustering and Feature Heatmap Display

The optimal number of clusters was determined based on the Calinski–Harabasz score. In this study, the optimal number of clusters for the T1CE and T2FLAIR sequences was identified as 3. Figure 3 presents a trinity case comparison of MRI habitat heatmaps, WSI heatmaps, and nomogram predictions between high‐risk and non‐high‐risk patients.

**FIGURE 3:**
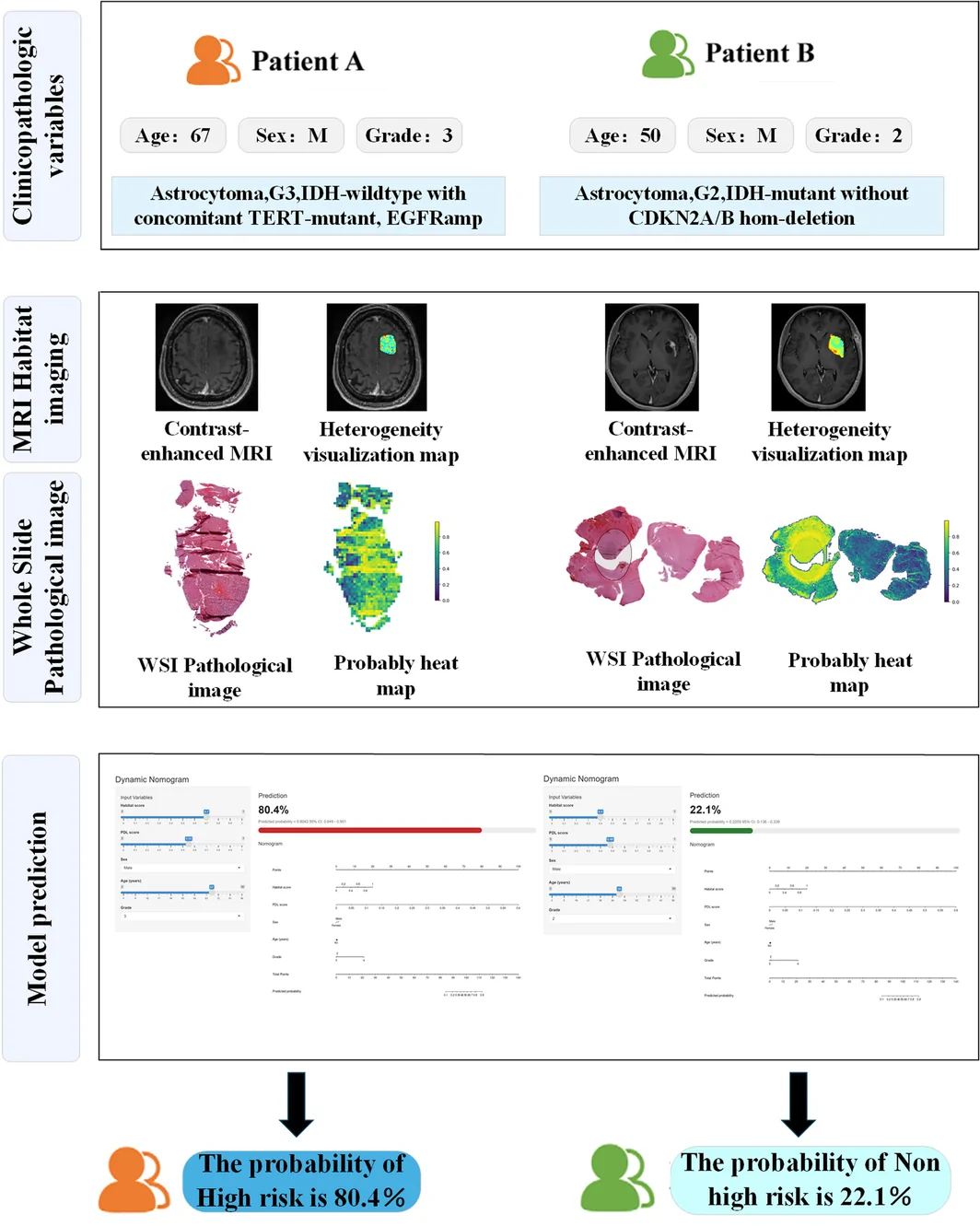
This figure presents a trinity visualization of two representative cases with high‐risk and non‐high‐risk phenotypes, integrating MRI habitat heatmaps, WSI heatmaps, and nomogram predictions.

### 3.3 Feature Extraction and Selection

A total of 10,458 habitat radiomic features and 512 deep learning pathomics features were extracted. Following the application of LASSO regression, we retained 20 habitat radiomic features and 19 deep learning pathomics features. Among the chosen habitat radiomic features, 12 originate from the T1CE sequence, while 8 come from the T2FLAIR sequence. The two sequences habitat radiomic features that made the most substantial contributions are T1C__intra_wavelet_HHL_glcm_Correlation_h1 and FLAIR__intra_logarithm_firstorder_RootMeanSquared_h2.

### 3.4 Evaluation of Model Prediction Performance

The ROC curves indicate that among the two single‐modality models, the PDL model achieved a better AUC value (Training set: 0.852, Test set: 0.821) compared to the Habitat model's AUC values (Training set: 0.832, Test set: 0.798). The Combined model demonstrated the best performance in the study cohort, with AUC values of 0.888 and 0.836 in the training and test sets, respectively (Figure 4). The calibration curves show that the Combined model has the best fit, indicating excellent calibration performance (Figure 5). The model performance table is shown in Supporting Information 7. Additionally, the decision curves reveal that the Combined model yields the highest net benefit, suggesting its good potential for clinical application (Figure 5).

**FIGURE 4:**
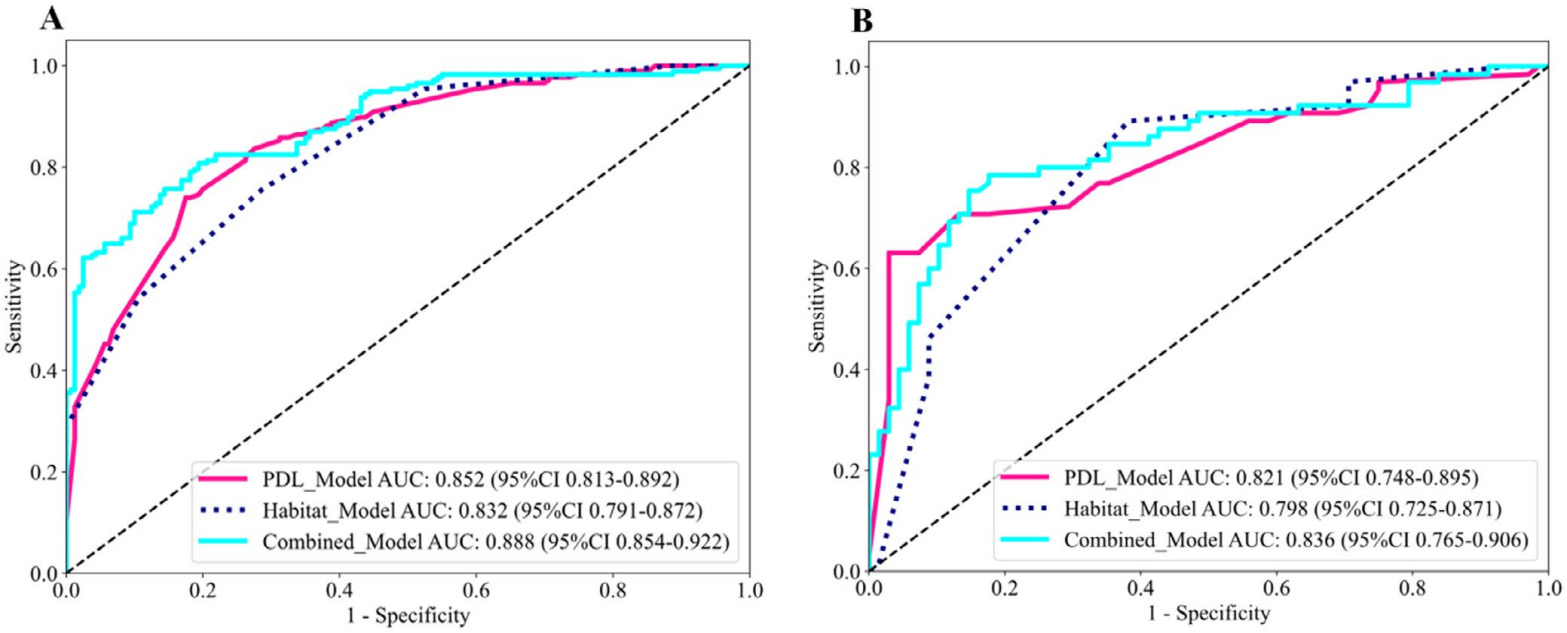
(A, B) respectively display the ROC curves of three models on the training set and the test set.

**FIGURE 5:**
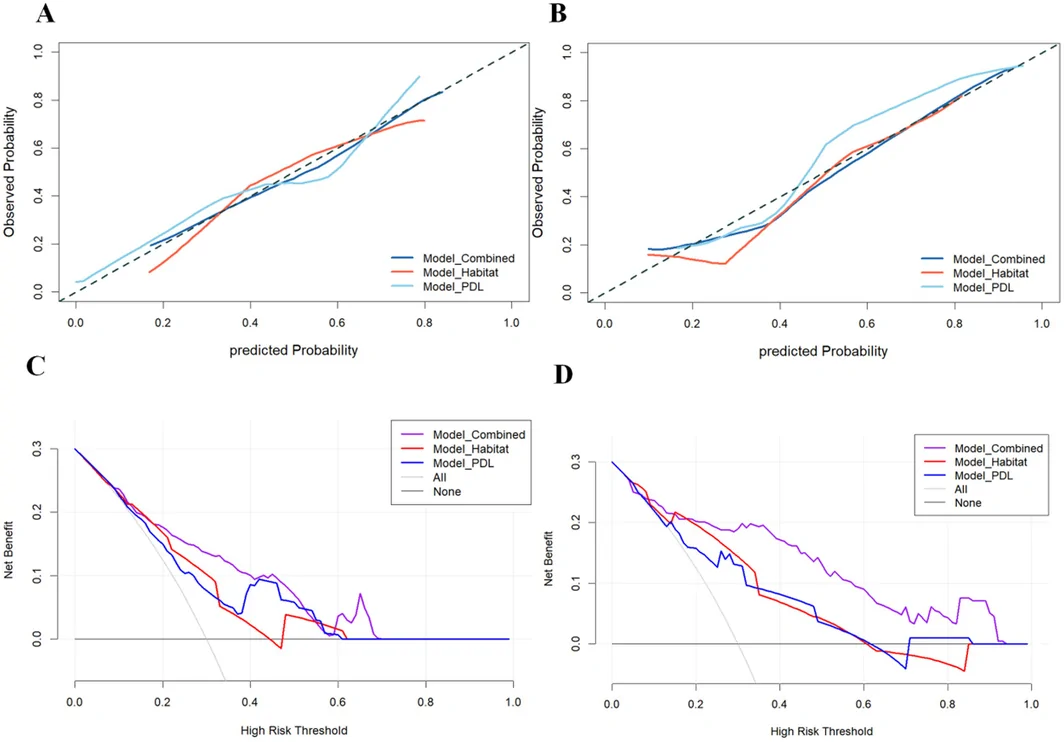
(A, B) respectively display the calibration curves of three models. The black diagonal line represents the ideal standard, and closer proximity to this line indicates better evaluation performance. (C, D) respectively show the decision curves of the three models, with the Combined model demonstrating the best net benefit.

### 3.5 Evaluation of the Prognostic Value of Risk Stratification by the Combined Model

By utilizing the output scores generated by the Combined model, we consistently categorized the study dataset into high‐risk and low‐risk groups. The findings from the Log‐rank test indicated that the OS in both the training and test sets could be significantly distinguished according to the scores assigned by the Combined model Supporting Information 5.

### 3.6 Transcriptomic Correlates of Model‐Based Risk Stratification

We conducted an exhaustive analysis of RNA‐seq data derived from the comparison between high‐risk and low‐risk groups, leading to the identification of 100 differentially expressed genes (DEGs). Among these DEGs, 81 exhibited significant upregulation, while 19 demonstrated significant downregulation. GO enrichment analysis showed that these genes were mainly involved in biological processes such as extracellular matrix organization, collagen fibril organization, and wound healing; cellular components including the collagen‐containing extracellular matrix and endoplasmic reticulum lumen; and molecular functions including extracellular matrix structural constituent and heparin binding. KEGG enrichment analysis further identified pathways including ECM–receptor interaction and focal adhesion, while the Wnt signaling pathway was also among the top 10 enriched KEGG pathways. Overall, the GO and KEGG results showed convergent patterns involving extracellular matrix‐ and cell–matrix interaction‐related biological themes. The enrichment results from GO and KEGG analyses are presented in Supporting Information 6.

## 4 Discussion

This study developed a Combined model integrating macroscopic MRI habitat imaging, microscopic WSI‐based pathomics, and clinical information to predict high‐risk molecular subtypes of gliomas. The model achieved strong performance, with AUCs of 0.888 in the training set and 0.836 in the test set. A dynamic nomogram was developed to visualize predictions, and the model output scores effectively stratified patients into high‐ and low‐risk groups with significant survival differences. Exploratory transcriptomic analysis further linked the risk stratification to transcriptional features potentially associated with glioma biology. Overall, this integrative framework offers a new perspective for quantitative assessment of glioma heterogeneity and may facilitate personalized treatment planning in clinical practice.

Recently, several studies have demonstrated the feasibility of using artificial intelligence techniques to predict integrated high‐risk molecular subtypes of gliomas [22, 23, 24]. Yang et al. [25], employed conventional MRI sequences, habitat imaging technology, and seven classifiers to predict high‐risk molecular subtypes of lower‐grade gliomas. Among them, the Extra Trees classifier exhibited the best predictive performance, achieving AUC values of 0.802, 0.771, and 0.768 in the training, test, and external validation sets, respectively. Wang et al. [26], utilized convolutional neural networks (CNNs) for deep learning to construct a comprehensive neuropathological classification model for adult diffuse gliomas based on WSIs, with AUC values exceeding 0.9. However, previous studies relied solely on either a single MRI or digital pathology images, which may not provide a more comprehensive set of macroscopic and microscopic features for predicting high‐risk molecular subtypes of gliomas. Based on the hypothesis that tumor biology determines treatment outcomes across different spatial scales (such as the individual, tissue, cellular, and molecular levels) [27], we explored currently available factors from glioma physical examinations, radiology, pathology, and clinical laboratories. We established a Combined model that incorporates MRI habitat imaging, digital pathology, and clinical information. The Combined model consistently demonstrated superior performance. This may be attributed to the complementary value demonstrated by clinical, habitat radiomics, and deep learning pathomics features in the preprocessing of individualized predictions. Specifically, habitat imaging technology conceptualizes the tumor as a dynamic ecosystem composed of multiple sub‐regions with distinct pathophysiological characteristics [28, 29]. By re‐segmenting tumor sub‐regions in multimodal MRI images and visualizing them based on radiomic semantic features, it enables the establishment of quantitative associations between macroscopic imaging features and microscopic cellular/molecular characteristics [30]. The integration of Transformer‐based deep learning in histopathology with MRI habitat imaging is complementary. Applying the Transformer network architecture to WSIs allows for superior capture of global contextual information, facilitating the in‐depth extraction of quantitative histopathological features significantly correlated with high‐risk molecular subtypes from a microscopic perspective. This approach reveals the intrinsic molecular signatures, gene expression patterns, and tumorigenic mechanisms underlying tumor development, thereby enhancing our understanding of the biological behavior and malignant transformation processes in gliomas [31]. By fusing these modalities across scales, we can effectively dissect tumor molecular heterogeneity and microenvironmental characteristics, which is pivotal for accurately predicting high‐risk molecular subtypes. This integrated framework holds significant promise for advancing precision oncology in glioma management. Despite the promising potential of habitat imaging, most previous studies, including ours, have primarily relied on conventional MRI sequences. Recently, Choi et al. [32] utilized multiparametric physiologic MRI‐based habitat analysis to distinguish molecular glioblastoma from histological glioblastoma, achieving an AUC of 0.87 with a habitat‐based multivariable model. Jeong et al. [33] further extended this approach to the longitudinal setting, correlating habitat and radiomic features with telomerase activity dynamics in recurrent IDH‐wildtype glioblastoma. Their multiparametric physiologic MRI‐based approach offers richer biological information, particularly in capturing perfusion and diffusion characteristics, whereas our T1CE/T2‐FLAIR‐based strategy is more limited in this regard.

The integration of multiple visualization approaches based on AI‐driven quantitative analysis is a notable highlight of this study. As is widely recognized, visualization plays a pivotal role in enhancing the interpretability of AI in medicine. For instance, Huang et al. [34] developed a dynamic nomogram interface based on a customized intratumoral heterogeneity (ITH) score to predict the probability of pathological complete response (pCR) in breast cancer in real time. Zheng et al. [35] utilized Grad‐CAM visualization heatmaps from a CT Transformer model to highlight image‐level differences associated with lymph node metastasis after neoadjuvant chemotherapy (NAC). In our study, we developed an online nomogram (https://gliomaresearch.shinyapps.io/GliomaNomogram/) as a practical clinical tool that allows clinicians to input key information and obtain individualized risk predictions. To further illustrate the model's utility, we also constructed a trinity case‐based visualization by integrating MRI habitat heatmaps, WSI heatmaps, and nomogram predictions for two representative patients (high‐risk vs. non‐high‐risk), enabling direct cross‐scale comparison from macroscopic imaging to microscopic pathology and finally to the predicted risk probability. This complementary approach combines a deployable prediction tool with an intuitive case‐based visualization and provides a comprehensive framework for interpreting glioma heterogeneity. We acknowledge, however, that the current reliance on WSI acquisition and deep learning inference limits the real‐time applicability of this nomogram in routine clinical practice. WSI scanning, image transmission, storage, and computational inference all require a substantial amount of time, which currently precludes the nomogram from serving as an intraoperative or point‐of‐care decision tool. We therefore reposition its clinical utility as more suitable for non‐urgent scenarios, such as comprehensive evaluation after surgery, multidisciplinary team discussions, and postoperative adjuvant treatment planning. With ongoing advances in whole‐slide scanning technology, edge computing, and lightweight transformer models, this time gap is expected to narrow considerably in the near future.

Another highlight of this study is the exploration of the underlying biological mechanisms based on patient risk stratification, which is conducted using the output scores of the Combined model. Recently, several studies have demonstrated the feasibility of multi‐omics research [36, 37, 38, 39, 40, 41]. Cheng et al. [42] developed a radiomics model based on preoperative CT images to predict the malignant potential of pancreatic cystic lesions. By integrating proteomic and lipidomic data from the cystic fluid, they explored the biological mechanisms, revealing that the radiomics scores might be driven by biological secretion and metabolic processes. Huang et al. [34] developed a radiomics model based on MRI features to predict the response to neoadjuvant chemotherapy in breast cancer. They also performed pathway enrichment analysis on the groups stratified by nomogram model scores, reflecting the essential differences in tumor biological behaviors among different groups. In this study, exploratory transcriptomic analysis provided complementary biological context for the Combined model‐derived risk stratification. GO and KEGG enrichment analyses of differentially expressed genes between risk groups consistently highlighted extracellular matrix remodeling and cell–matrix interactions, including ECM organization, collagen‐related components, ECM‐receptor interaction, and focal adhesion. These convergent findings suggest that ECM‐related processes may represent transcriptional features associated with model‐derived risk states and provide biological support for the model‐based stratification.

This study also has certain limitations. Firstly, it is a retrospective study. Due to the limited sample size, we will further collect large‐scale sample data from more medical centers to conduct prospective studies, aiming to validate the generalization ability and accuracy of the constructed model. Secondly, although standardized image registration and isotropic resampling were performed, additional harmonization or scanner–/center‐specific analyses, such as ComBat correction and stratified analyses, were not conducted. Residual inter‐center and inter‐scanner variability may therefore have affected radiomic features, particularly texture‐related features, and potentially reduced their reproducibility and model generalizability. Future multicenter studies incorporating dedicated harmonization strategies are warranted. Thirdly, given the infiltrative growth pattern of gliomas, transitional states may exist between tumor habitats. The hard‐assignment strategy of K‐means clustering may therefore oversimplify this continuous heterogeneity. Future studies using soft/probabilistic or spatially informed clustering approaches may better characterize these transitional states. In addition, the genomic and transcriptomic analyses were both performed on TCGA data, which has a relatively small sample size and was collected over a wide time span, and the risk stratification may partially reflect known molecular and grade differences; therefore, the biological interpretations should be considered preliminary.

In conclusion, this study integrated macroscopic MRI habitat imaging, microscopic WSI‐based pathomics, and clinical information to construct a cross‐scale predictive model for high‐risk molecular subtypes of gliomas. The model output scores enabled effective patient risk stratification. In addition, exploratory transcriptomic analysis linked the model‐based risk stratification to transcriptional features related to extracellular matrix remodeling and cell–matrix interactions, providing complementary biological context for the model predictions. This framework offers a new perspective for quantitative assessment of glioma heterogeneity and may facilitate early warning of malignant transformation and personalized treatment planning in clinical practice.

## Author Contributions

W.N. and H.Z. conceived and designed the study. X.D., X.L., Z.L., Q.L., X.Y., Y.T. and X.W. collected the data. W.N.developed and validated the deep learning networks. W.N., X.D. and X.L. accessed and verified the underlying data. W.N., X.D., and X.L. wrote the original manuscript. G.Y. and H.Z. reviewed and edited the manuscript. All authors read and approved the final manuscript. All authors had full access to the data in the study and take responsibility for the integrity of the data and the accuracy of the data analysis, as well as the decision to submit for publication.

## Funding

This work was supported by the National Natural Science Foundation of China (U21A20386, 82371941) and the Basic Research Project of Shanxi Province (202303021211204).

## Ethics Statement

The study was conducted in accordance with the Declaration of Helsinki and received ethical approval from the Institutional Review Board of Shanxi Medical University (Approval No. 2021‐K‐K073), with participation from three affiliated hospitals: The First Hospital of Shanxi Medical University, Shanxi Provincial People's Hospital, and Shanxi Bethune Hospital. Due to the retrospective nature of the study and anonymized data processing, the requirement for informed consent was waived.

## Conflicts of Interest

The authors declare no conflicts of interest.

## Supporting information


**Supporting Information: 1** List of patients included from TCGA.
**Supporting Information:** 2 Detailed scanning protocol for MRI.
MRI scanning protocol in the training set(First Hospital of Shanxi Medical University, Shanxi Provincial People's Hospital, Shanxi Bethune Hospital).
**Supporting Information:** 3 The flowchart for patient selection.
**Supporting Information:** 4 Architecture diagram of the Vision Transformer (ViT).
**Supporting Information:** 5 Kaplan–Meier curves for the training set and the test set.
**Supporting Information:** 6 The enrichment results from GO and KEGG analyses.
**Supporting Information:** 7 Performance of the predictive models in the study sets.

## Data Availability

The image data from the TCGA public database, as a test set, can be obtained at https://www.cancerimagingarchive.net/. Clinical information of patients can be accessed at https://www.cancer.gov/ccg/research/genome‐sequencing/tcga. The specific patient IDs included in this study are detailed in the Supporting Information 1. The data from the training set cannot be publicly released due to patient privacy concerns but can be obtained from the corresponding author upon reasonable request approved by the institutional review board of all enrolled centers. The code for image preprocessing (including MRI and WSI), habitat imaging analysis, deep learning model training, and machine learning‐based modeling is available at the OnekeyAI platform (https://github.com/OnekeyAI‐Platform/onekey).
