# Supplementary material for: Integrating Multimodal MRI Habitat and Transformer‐Based Pathomics to Predict High‐Risk Molecular Subtypes and Explore Biological Mechanisms in Adult Diffuse Gliomas
Source: CNS Neurosci Ther. 2026 Sep 28;32(10):e71171. doi: 10.1002/cns.71171 (PMC13618620; doi:10.1002/cns.71171)
Supplement: Supplementary file 1 [file CNS-32-e71171-s001.docx]

**Supplementary Information**

**Supplementary Information(1):Tabel. S1.List of patients included from TCGA**

| TCGA-CS-4941 | TCGA-DU-5855 | TCGA-DU-7019 | TCGA-DU-A5TS | TCGA-FG-8189 | TCGA-HT-7692 | TCGA-HT-A5RC |
| --- | --- | --- | --- | --- | --- | --- |
| TCGA-CS-4942 | TCGA-DU-5871 | TCGA-DU-7294 | TCGA-DU-A5TT | TCGA-FG-A4MT | TCGA-HT-7693 | TCGA-HT-A61A |
| TCGA-CS-4943 | TCGA-DU-5872 | TCGA-DU-7298 | TCGA-DU-A5TU | TCGA-FG-A4MU | TCGA-HT-7694 | TCGA-02-0047 |
| TCGA-CS-4944 | TCGA-DU-5874 | TCGA-DU-7299 | TCGA-DU-A5TW | TCGA-FG-A6IZ | TCGA-HT-7855 | TCGA-06-0130 |
| TCGA-CS-5393 | TCGA-DU-6397 | TCGA-DU-7300 | TCGA-DU-A5TY | TCGA-FG-A6J1 | TCGA-HT-7856 | TCGA-06-0139 |
| TCGA-CS-5395 | TCGA-DU-6399 | TCGA-DU-7301 | TCGA-DU-A6S2 | TCGA-FG-A60K | TCGA-HT-7860 | TCGA-06-0878 |
| TCGA-CS-5396 | TCGA-DU-6400 | TCGA-DU-7302 | TCGA-DU-A6S3 | TCGA-FG-A87N | TCGA-HT-7874 | TCGA-06-5408 |
| TCGA-CS-5397 | TCGA-DU-6401 | TCGA-DU-7304 | TCGA-DU-A6S6 | TCGA-FG-A713 | TCGA-HT-7877 | TCGA-06-5412 |
| TCGA-CS-6186 | TCGA-DU-6404 | TCGA-DU-7306 | TCGA-DU-A6S7 | TCGA-HT-7473 | TCGA-HT-7879 | TCGA-06-5413 |
| TCGA-CS-6188 | TCGA-DU-6405 | TCGA-DU-7309 | TCGA-DU-A6S8 | TCGA-HT-7475 | TCGA-HT-7881 | TCGA-12-3650 |
| TCGA-CS-6290 | TCGA-DU-6407 | TCGA-DU-8162 | TCGA-EZ-7264 | TCGA-HT-7602 | TCGA-HT-7882 | TCGA-14-0789 |
| TCGA-CS-6666 | TCGA-DU-6408 | TCGA-DU-8163 | TCGA-FG-5962 | TCGA-HT-7604 | TCGA-HT-7884 | TCGA-14-0790 |
| TCGA-CS-6667 | TCGA-DU-6410 | TCGA-DU-8164 | TCGA-FG-5964 | TCGA-HT-7605 | TCGA-HT-8018 | TCGA-14-0871 |
| TCGA-CS-6668 | TCGA-DU-7008 | TCGA-DU-8165 | TCGA-FG-6688 | TCGA-HT-7608 | TCGA-HT-8105 | TCGA-14-1823 |
| TCGA-CS-6670 | TCGA-DU-7010 | TCGA-DU-8166 | TCGA-FG-6690 | TCGA-HT-7616 | TCGA-HT-8107 | TCGA-14-1829 |
| TCGA-DU-5849 | TCGA-DU-7013 | TCGA-DU-8167 | TCGA-FG-6691 | TCGA-HT-7680 | TCGA-HT-8113 | TCGA-19-1390 |
| TCGA-DU-5852 | TCGA-DU-7014 | TCGA-DU-8168 | TCGA-FG-6692 | TCGA-HT-7684 | TCGA-HT-8114 | TCGA-19-2624 |
| TCGA-DU-5853 | TCGA-DU-7015 | TCGA-DU-A5TP | TCGA-FG-7637 | TCGA-HT-7686 | TCGA-HT-8563 | TCGA-19-5960 |
| TCGA-DU-5854 | TCGA-DU-7018 | TCGA-DU-A5TR | TCGA-FG-7643 | TCGA-HT-7690 | TCGA-HT-A5R7 | TCGA-76-4926 |

**Supplementary Information(2):Detailed scanning protocol for MRI**

**MRI scanning protocol in the training set(First Hospital of Shanxi Medical University, Shanxi Provincial People's Hospital, Shanxi Bethune Hospital):**

First Hospital of Shanxi Medical University: Preoperative MRI scans were conducted utilizing a 3.0-Tscanner (Signa HDxt, GE Healthcare, USA) equippedwith an eight-channel array coil. The acquisition protocol included CE-T1WI (repetition time/echo time, 195 ms/4.76 ms; field of view, 240mm; thickness/slice interval, 5.0 mm/1.5 mm; and matrix, 256 × 256) and T2-FLAIR (repetition time/echo time, 8000 ms/95 ms; field of view, 240 mm; thickness/slice interval, 5.0 mm/1.5 mm; and matrix, 256 × 256). CE-T1WI was obtained after the injection of 0.1 mmol/kg of gadolinium chelate contrast medium.

Shanxi Provincial People's Hospital: MRI scanning was performed using an 8-channel phased array coil on a 3.0 T Scanner (Discovery 750, GE Healthcare, USA). The acquisition protocol encompassed both CE-T1 sequence (TR/TE: 195 ms/4.76 ms; FOV: 240 mm×240 mm; slice thickness/slice gap: 5.0 mm/1.5 mm; matrix: 256×256) and T2-FLAIR sequence (TR/TE: 8000 ms/95 ms; FOV: 240 mm×240 mm; slice thickness/slice gap: 5.0 mm/1.5 mm; matrix: 256×256).

Shanxi Bethune Hospital: The acquisition protocol of the Siemens Skyra 3.0T MRI are as follows:For the CE-T1WI acquisition protocol: TR/TE 2000ms / 42ms, Field of view (FOV) 230mm, Slice thickness/Slice gap 5.5mm/1.3mm, Matrix 256×256. All T1-enhanced scans use gadopentetate dimeglumine injection, administered at a dose of 0.1mmol/kg, injected through the elbow vein at a rate of 2-3ml/s.For the T2-FLAIR acquisition protocol: TR/TE 8000ms / 88ms, Slice thickness/Slice interval, 5.5mm/1.3mm, FOV 230mm, Matrix, 256×256.

**Supplementary Information(3):The flowchart for patient selection.**

**Supplementary Information(4): Architecture diagram of the Vision Transformer (ViT) .**

The input image(in the upper left corner) is divided into fixed-size non-overlapping patches, each of which is flattened into a vector. A fully connected layer is then used to project the vector of each patch into the model's dimension, resulting in Patch Embeddings.A learnable positional encoding is added to each Patch Embedding, and a learnable classification token is appended to the beginning of the sequence (represented by the numbered two-color rectangle in the diagram).The Patch Embeddings, now including positional encoding and the classification token, are fed into the Transformer Encoder for feature extraction and contextual modeling.The classification head (MLP Head) takes the output vector corresponding to the [class] Embedding and maps it to the category space through a multilayer perceptron (MLP), completing the image classification task.

**Supplementary Information(5): Kaplan-Meier curves for the training set and the test set**

A and B respectively represent the high-risk and low-risk groups divided based on the output scores thresholds of the Combined model from the training set. The blue line indicates the low-risk group, while the yellow line indicates the high-risk group. The results show that the p-values for both the training set (A) and the test set (B) are less than 0.05, indicating statistically significant differences between the groups.

**Supplementary Information(6): The enrichment results from GO and KEGG analyses**

GO and KEGG enrichment analyses of differentially expressed genes between the high- and low-risk groups. (A–C) GO enrichment analyses showing the top 10 enriched terms in Biological Process (BP), Cellular Component (CC), and Molecular Function (MF), respectively; (D) KEGG enrichment analysis showing the top 10 enriched pathways.

**Supplementary Information(7): Performances of the predictive models in the study sets.**

| **Model and metric** | **Accuracy** | **AUC（95%CI）** | **Sensitivity** | **Specificity** | **PPV** | **NPV** |
| --- | --- | --- | --- | --- | --- | --- |
| **Training set** |  |  |  |  |  |  |
| PDL-Model | 0.757 | 0.852（0.8126-0.8922） | 0.684 | 0.837 | 0.823 | 0.705 |
| Habitat-Model | 0.709 | 0.832（0.7915-0.8725） | 0.542 | 0.894 | 0.850 | 0.638 |
| Combined-Model | 0.804 | 0.888（0.8538-0.9217） | 0.802 | 0.806 | 0.821 | 0.787 |
| **Test set** |  |  |  |  |  |  |
| PDL-Model | 0.782 | 0.821（0.7481-0.8949） | 0.585 | 0.971 | 0.950 | 0.710 |
| Habitat-Model | 0.692 | 0.798（0.7248-0.8714） | 0.462 | 0.912 | 0.833 | 0.639 |
| Combined-Model | 0.797 | 0.836（0.7654-0.9063） | 0.769 | 0.824 | 0.806 | 0.789 |
